# Chronic disease in the Mojave desert tortoise: Host physiology and recrudescence obscure patterns of pathogen transmission

**DOI:** 10.1002/ece3.3480

**Published:** 2017-11-04

**Authors:** Franziska C. Sandmeier, K. Nichole Maloney, C. Richard Tracy, David Hyde, Hamid Mohammadpour, Ron Marlow, Sally DuPré, Kenneth Hunter

**Affiliations:** ^1^ Biology Department Colorado State University – Pueblo Pueblo CO USA; ^2^ Department of Pathology, Microbiology, and Immunology Vanderbilt University Nashville TN USA; ^3^ Biology Department University of Nevada Reno Reno NV USA; ^4^ Department of Microbiology and Immunology University of Nevada Reno Reno NV USA

**Keywords:** ectotherm, immune function, reptile, subclinical disease, wildlife disease

## Abstract

A seminatural, factorial‐design experiment was used to quantify dynamics of the pathogen *Mycoplasma agassizii* and upper respiratory tract disease in the Mojave desert tortoise (*Gopherus agassizii*) over 2 years. Groups of initially healthy animals were separated into serologically positive (seropositive), seronegative, and artificially infected groups and paired into 23 pens. We found no evidence of long‐term immune protection to *M. agassizii* or of immunological memory. Initially seronegative, healthy tortoises experienced an equal amount of disease when paired with other seronegative groups as when paired with seropositive and artificially infected groups—suggesting that recrudescence is as significant as transmission in introducing disease in individuals in this host–pathogen system. Artificially infected groups of tortoises showed reduced levels of morbidity when paired with initially seronegative animals—suggesting either a dilution effect or a strong effect of pathogen load in this system. Physiological dynamics within the host appear to be instrumental in producing morbidity, recrudescence, and infectiousness, and thus of population‐level dynamics. We suggest new avenues for studying diseases in long‐lived ectothermic vertebrates and a shift in modeling such diseases.

## 1 INTRODUCTION

Recrudescent diseases have predominantly been studied in humans—predominantly on a molecular level in certain viral diseases, in immunosuppressed individuals, or in cases where drug resistance can evolve (e.g., the varicella‐zoster virus which can result in chicken pox and shingles, infections in HIV‐positive patients, malaria) (Okoro et al., 2012; Rentier et al., 1996; Slater, Griffin, Ghani, & Okell, 2016). In wildlife populations, the explicit study of recrudescent diseases and the population dynamics of such diseases are rare. The study by Wang et al. (2013) is a notable exception, and they found that the role of recrudescence in the maintenance of viral infection in flying foxes (*Pteros* species) is biologically significant and warrants more attention. Recrudescence may be particularly influential in disease persistence in long‐lived host species, especially when transmission rates are low (Slater et al., 2016; Wang et al., 2013). Furthermore, if transmission rates are high, such as in dense populations, then recrudescence can lead to intensified disease outbreaks in the absence of other perturbations (e.g., Slater et al., 2016). Despite its importance in disease dynamics, recrudescence has been understudied in comparison with transmission of pathogens—possibly due to the longer ecological time frame necessary to quantify recrudescence.

**Figure 1:**
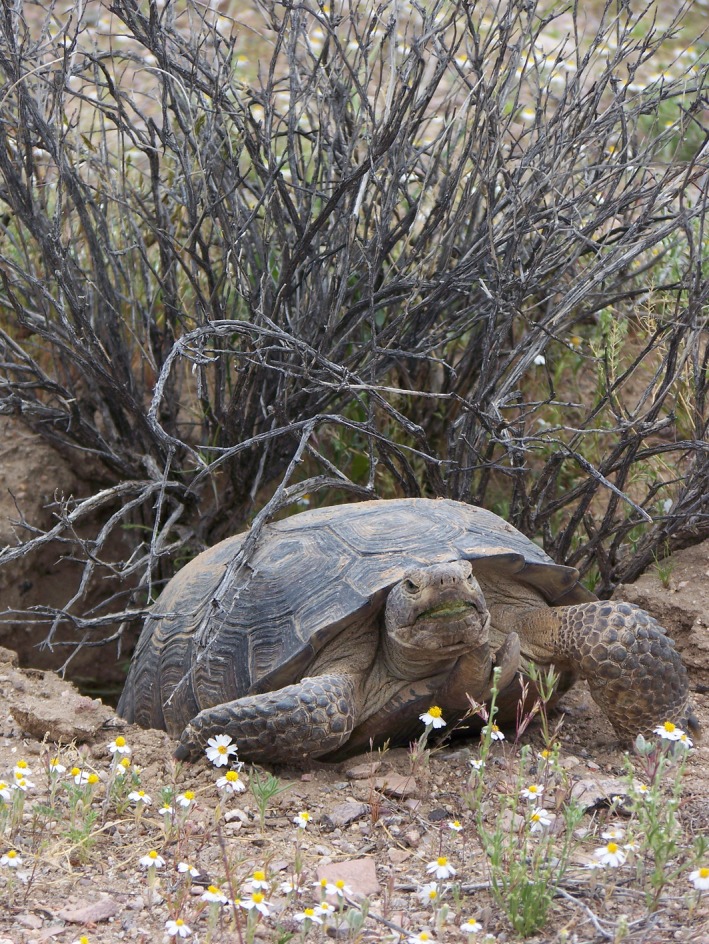
A wild Mojave desert tortoise (*Gopherus agassizii*), emerging from a typical burrow, at the base of a creosote shrub

Here, we quantify the role of recrudescence and transmission of *Mycoplasma agassizii*, a cause of upper respiratory tract disease (URTD) in Mojave desert tortoises (*Gopherus agassizii*) (Figure 1; Schumacher, Brown, Jacobson, Collins, & Klein, 1993; Brown et al., 1994). Previous research has focused on the short‐term etiology of *M. agassizii* within hosts in laboratory settings, the distribution of *M. agassizii* across the range of the Mojave desert tortoise, and the quantification of transmission between diseased (moribund) and healthy tortoises in seminatural conditions (Aiello et al., 2016; Brown et al., 1994; Sandmeier et al., 2013; Schumacher et al. 1993). While mild URTD rates range from 0% to 15% on average within populations, recent evidence by quantitative PCR suggests that 20–85% of individuals within these populations may harbor subclinical infections—with very low loads of *M. agassizii* (Braun et al., 2014; Chava Weitzman, unpublished data; Sandmeier et al., 2013). Therefore, the possibility exists that URTD, as a disease, emerges due to a combination of recrudescence and transmission.

Here, we provide evidence from a 2‐year‐long, seminatural transmission experiment conducted on healthy, previously wild animals with and without antibody responses to *M. agassizii*. Because disease progresses slowly in these animals, the long time period of this study was crucial to capture disease dynamics within both individuals and groups of tortoises that has not been evident in earlier studies (Brown et al., 1994; Schumacher et al., 1993). A total of 276 tortoises without signs of disease were classified as (1) seronegative, (2) seronegative and experimentally infected with *M. agassizii*, and (3) seropositive (six individuals per group). Groups were placed into 23 pens to create pairs of treatments: seronegative–seronegative, seronegative–infected, seronegative–seropositive, and infected–seropositive. This experiment allowed us to test hypotheses concerning the natural progression of disease both in individuals and in groups of individuals, modeling what would occur in natural populations (albeit at higher densities than typically occur in nature). The original focus of the experiment was on transmission, and seropositive–seropositive or infected–infected pens were included in the study.

All tortoises were adults, originally removed from development sites in the Las Vegas Valley, where *M. agassizii* is widespread and tortoise populations are relatively dense (Sandmeier et al., 2013). Although sensitive genetic tests to assess the presence of pathogen in tortoises did not yet exist at the time of this experiment, most of these animals were likely exposed to the mycoplasma at some point in their lives, and some animals within each experimental group likely harbored low levels of *M. agassizii*. Antibody responses of desert tortoises, even to nonreplicating antigens, are known to remain elevated for more than a year postinfection, and evidence suggests that antibody responses are usually stimulated by high loads of *M. agassizii* (Aiello et al., 2016; Sandmeier, Tracy, DuPré, & Hunter, 2012). Thus, the seropositive tortoises likely had a significant enough infection to make an antibody response in the recent past and, at that time, may have been more susceptible to *M. agassizii* than other tortoises. Therefore, we assumed that seropositive animals were more likely to harbor subclinical infections at the beginning of the experiment than seronegative animals. Clearance rates for *M. agassizii* are unknown and are likely negligible, as is the case for other chronic mycoplasmal infections of vertebrates (Razin, Yogeve, & Naot, 1998; Rottem, 2003; Simecka et al., 1993). Equally important to the analysis of this study is that severe disease results in the shedding of visible, external exudate, which is associated with high loads of *M. agassizii*, and is thought to be necessary for an animal to transmit disease (Aiello et al., 2016).

First, we quantified the progression of URTD and antibody responses within individuals—including the length of time until seroconversion and the longevity of antibody responses. We tested two hypotheses pertaining to individual antibody responses. (1) Artificially infected, seronegative animals will acquire disease at a greater rate than seronegative animals that were not infected with *M. agassizii*. (2) Tortoises do not exhibit clear immunological memory to *M. agassiziii*. We tested an additional three hypotheses pertaining to the dynamics of disease in groups of animals. (3) Seronegative groups of animals will exhibit signs of URTD at a greater rate when paired with seropositive or infected groups of animals than when paired with other seronegative animals. (4) At the end of the 2‐year duration of the experiment, the number of animals in each group that seroconvert or show severe signs of URTD (visible exudate) should be associated with some combination of measured variables (e.g., initial antibody and infection status, exposure to other groups of animals, and loss in mass). (5) Among groups of infected animals, rates of disease will be less when individuals are paired with seronegative individuals than when paired with seropositive individuals (dilution effect). Because serology and URTD are known to be decoupled within individuals (Braun et al., 2014; Sandmeier et al., 2013, 2017), our focus was on group‐level measures of disease.

## 2 MATERIALS AND METHODS

### 2.1 Seminatural enclosures

Adult tortoises were selected from animals displaced within the Las Vegas valley prior to 2003 and held at the Desert Tortoise Conservation Center in Las Vegas, Nevada, USA. All animals were of wild origin and selected to be in good body condition without any signs of URTD. Tortoises were then sorted into groups of six tortoises (four females and two males in each group): (1) seronegative, (2) seronegative and artificially infected with *M. agassizii*, and (3) seropositive. Tortoises were assigned to groups to assure similar body size distributions among pens. Because antibody levels are known to exhibit seasonal fluctuations, more than 276 tortoises were repeatedly tested by an ELISA (Schumacher et al., 1993) by the Desert Tortoise Conservation Center throughout spring and summer of 2003, and tortoises with ambiguous antibody results were excluded from the study. Tortoises with two positive or two negative antibody tests prior to October 2003 were considered seropositive or seronegative, respectively. To infect tortoises artificially, 0.5 ml of 10^8^ cells of *M. agassizii* strain PS6 (ATTC 700616) in SP4 broth was injected into the nares with sterile, disposable syringes. All other tortoises received an injection of an equal volume of sterile SP4 broth as a procedural control.

Tortoise groups were paired in 23 pens (five seronegative/seronegative, six seronegative/seropositive, six seronegative/infected, and six seropositive/infected). Pens were constructed of 1.75 cm hardware cloth, 80 × 90 m in size, with a 2 m alley between adjacent pens. Pens enclosed native vegetation, and animals were provided with artificial burrows. On a monthly basis (October 2003–September 2005), accessible tortoises (in the open or less than a meter deep in burrows) were examined for signs of URTD and had 0.5–1.0 ml blood drawn via brachial venipuncture.

### 2.2 Serology

Tortoise samples initially collected by the Desert Tortoise Conservation Center were tested at the University of Florida (Gainesville, FL) at least two times (as detailed above) by a monoclonal enzyme‐linked immunosorbent assay (ELISA), specific to the light chain common to all tortoise antibodies or isotypes of immunoglobulins (Ig) (Schumacher et al., 1993). For subsequent ELISA testing, we used a polyclonal, total Ig ELISA (Hunter, DuPre, Sharp, Sandmeier, & Tracy, 2008). ELISAs were run on one blood sample from each tortoise during the following seasons: fall 2003, winter 2003, spring 2004, fall 2004, spring 2005, and late summer 2005. These times were chosen both to detect early seroconversion by infected animals in 2003/2004 and to reflect subsequent sampling times during which blood was collected from most animals (e.g., animals not too deep in burrows to sample). A threefold increase in antibody titer was taken to indicate seroconversion, and a threefold decrease in antibody titers of seropositive animals was interpreted as a return to pre‐infection antibody levels (Origgi, 2007). Both initial antibody status and eventual seroconversion of animals were verified in a randomly selected group of animals with a confirmatory Western blot and/or isotype‐specific (IgM, IgY) polyclonal ELISAs (Hunter et al., 2008; Mohammadpour, 2011). Western blots measure the relative numbers of different types of antibodies produced, and such an increase in types of antibodies—as well as the production of IgY—is one of the hallmarks of an induced immune response (Murphy, 2014).

### 2.3 Polyclonal ELISA

Polyclonal ELISAs were conducted according to methods detailed in Hunter et al. (2008) and Sandmeier et al. (2013). Briefly, 50 μl of antigen containing 10 μg/ml *M. agassizii*, PS6 strain (Rockville, MD: ATCC 700616), in PBS was used to coat 96‐well plates (Immulon NUNC; Fisher Scientific, Fairlawn, NJ). Plates were incubated overnight at 4°C and then blocked with 5% nonfat milk in PBS for 2 hr at 4°C. Plates were washed four times with PBS‐Tween (PBST) after each subsequent incubation. Tortoise plasma (50 μl; serially diluted from 1:100 to 1:100,000) was added to each well and incubated overnight at 4°C. Polyclonal rabbit antitortoise antibody (50 μl, 1:10,000) was added and incubated at room temperature for 1 hr, followed by goat antirabbit IgG conjugated to horseradish peroxide (50 μl, 1:5,000; Zymed, San Francisco, CA) and incubated for 1 hr. Plates were developed with TMB microwell peroxidase substrate (KPL, Gaithersburg, ML), followed by 1N hydrochloric acid, with optical density determined at 450 nm.

### 2.4 Isotype‐specific ELISAs

All methods were similar to those for the total antibody ELISA, with the exception of the secondary antibodies (isotype‐specific IgM or IgY), described in Mohammadpour (2011).

### 2.5 Western blots

Western blots were performed according to Hunter et al. (2008). Protein from 20 μl *M. agassizii* antigen (25 μg/ml) was separated by gel electrophoresis using 10% Tris‐SDS for 53 min at 180 volts and transferred to nitrocellulose paper (Bio‐Rad, Hercules, CA) for 40 min at 80 volts. Nitrocellulose paper was blocked in TBS (5% nonfat dry milk) for 60 min. TBST (5% nonfat milk) was used to dilute all Igs, and TBST was used in washes between incubations. Nitrocellulose was incubated overnight at 4°C in 7 ml of tortoise plasma (1:100), followed by a 1‐hr incubation with rabbit antitortoise Ig (1:5,000) and goat antirabbit IgG conjugated to horseradish peroxidase (1:5,000; Zymed, San Francisco, CA), and developed with metal‐enhanced DAB (Thermo Scientific, Rockford, IL).

### 2.6 Visible disease

Animals were examined for visible signs of URTD monthly. URTD was defined as any indication of past or present discharge of mucous from the nares: partially or fully occluded nares, moisture or caked soil around the nares, and visible or external exudate (Braun et al., 2014; Sandmeier et al. 2017). If animals did have external discharge of exudate, it was classified as purulent (opaque) or serous (clear), and is referred to as “exudate” throughout the study. Because serous exudate often preceded purulent exudate, but animals showed intermittent and inconsistent levels of both, serous and purulent exudate were lumped together in statistical analyses.

### 2.7 Statistical analyses

**Figure 2:**
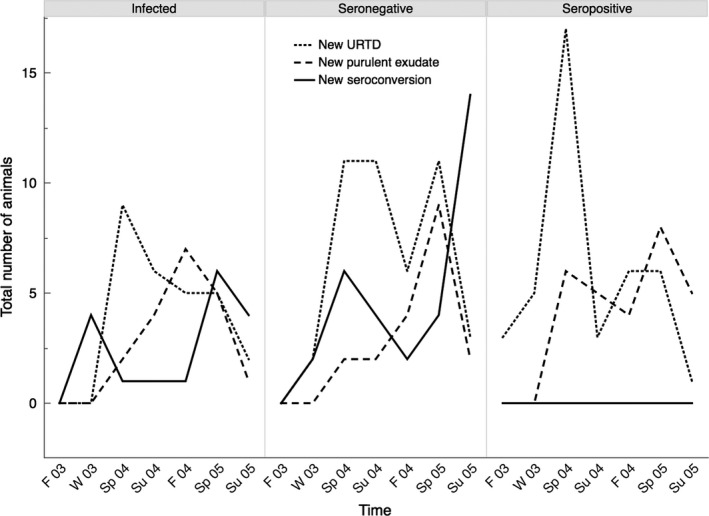
Total number of animals developing signs of URTD, developing purulent exudate, and seroconverting. The three panels show separate results for artificially infected (*n* = 72), initially seronegative (*n* = 102), and initially seropositive animals (*n* = 72). Seroconversions were not measured in Summer 2004. Seasons are abbreviated F (fall), W (winter), Sp (spring), and Su (summer), with the corresponding last two digits of the year (2003–2005)

**Figure 3:**
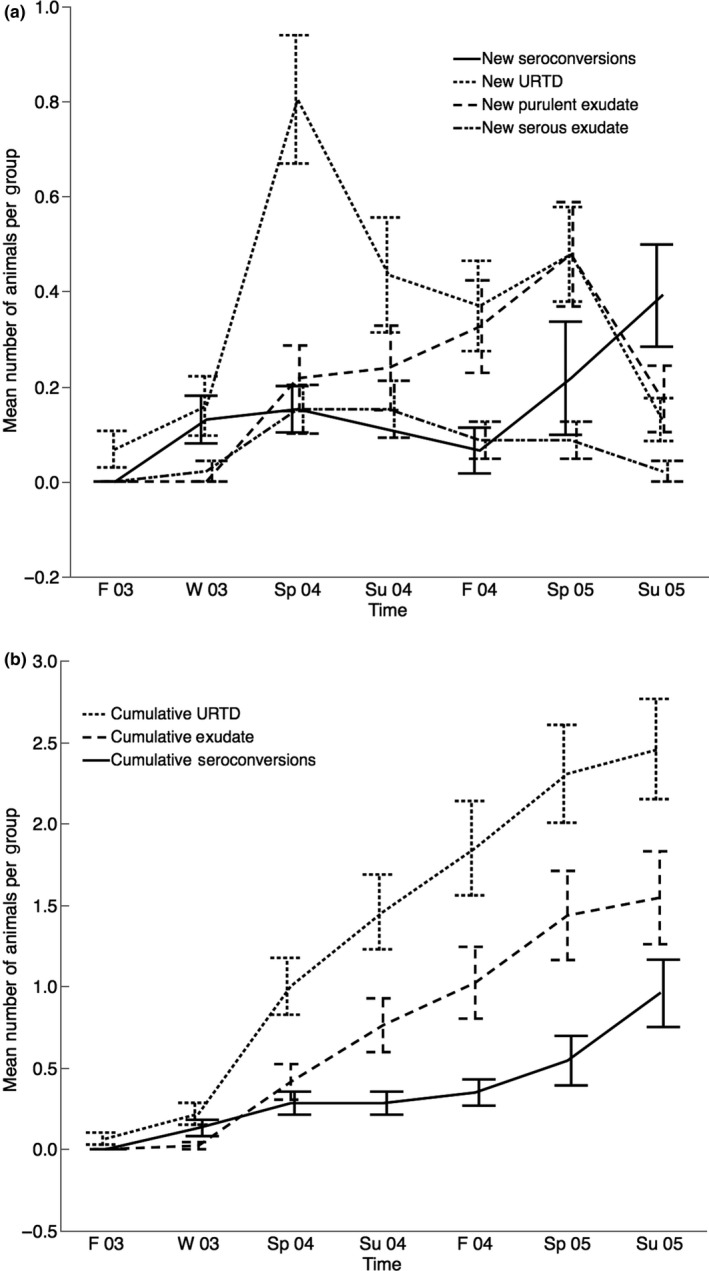
Mean number of animals per group (mean ± standard error; *n* = 46 groups, with each group consisting of six animals) showing (a) new signs of disease (URTD, purulent exudate, seroconversion) per season and (b) cumulative signs of disease (URTD, purulent exudate, and seroconversions) per season. Seroconversions were not calculated for Summer 2004. Seasons are abbreviated F (fall), W (winter), Sp (spring), and Su (summer), with the corresponding last two digits of the year (2003–2005)

In the following analyses, significant ANOVAs were followed by a Tukey's pairwise comparison to discern differences among groups. We used an alpha of 0.05 for all analyses, performed in JMP Pro 10.0.2 (SAS Institute Inc.). For any particular dataset analyzed, we restricted multiple comparisons as much as possible but analyzed the three main indicators of disease (any signs of URTD, visible discharge of exudate, and seroconversions) separately. These were usually separated in time and appear to represent the natural, but not inevitable, progression of disease (Figures 2 and 3).

### 2.8 Patterns of disease and antibody responses

We used an ANOVA to compare starting antibody titers (total Ig, IgY, and IgM) in a randomly selected subset of animals designated as seropositive or seronegative by the Desert Tortoise Conservation Center, in spring and summer of 2003. ELISA titers were fourth root transformed for normality. The progression of disease was qualitatively evaluated for both individuals and experimental groups of individuals (Figures 2 and 3). A contingency table analysis was used to compare the time of seroconversion between groups of seronegative and infected animals. We used a chi‐squared analysis to test for differences in seroconversion rates among males and females. Because a distinct peak in URTD was seen in spring of 2004 for seropositive groups, an ANOVA was used to compare numbers of animals per group who had developed URTD during this first period of the tortoise active season in the experiment.

To compare the magnitude of antibody responses, increases in total antibody titers were compared via ANOVA among three groups of animals: those with a primary response to *M. agassizii*, those with a secondary response to *M. agassizii* (seropositive animals with a clear secondary increase in antibody titers), and laboratory animals making a primary response to the nonreplicating antigen, ovalbumin, reported in Sandmeier et al. (2012). This laboratory study used the same serological technique, including the same negative controls, and therefore, the magnitude of antibody responses to different antigens (ovalbumin and *M. agassizii*) could be compared between these two studies (Sandmeier et al., 2012).

### 2.9 Disease transmission and recrudescence in experimental groups

For each experimental group of six animals, the number of animals with URTD, serous and purulent exudate, the number that seroconverted, and mean change in body mass were summed up per season. Depending on the analysis, data were square‐root‐transformed for normality. ANOVAs were used to compare change in body mass among groups of seronegative, infected, and seropositive tortoises. ANOVAs were used to compare the number of animals with URTD, exudate, and seroconversions in seronegative groups by treatment—paired with seronegative, infected, or seropositive groups. An ANOVA was used to compare the number of animals developing exudate at any point in the experiment among treatments in pens: seronegative/seronegative, seronegative/seropositive, seronegative/infected, and seropositive/infected.

Forward and backward selection of multiple, linear regression models—including assessment of all univariate models—was used to assess the best variables to predict number of animals seroconverting or acquiring signs of exudate. AICc scores and Akaike weights were used to compare models (Burnham & Anderson, 2002; Table 1). Correlated variables were not included in the same models (Table 1).

**Table 1:** Akaike information criteria and Akaike weights for the best models of (a) the number of animals seroconverting per experimental group and (b) the number developing visible exudate (discharge) from their nares throughout the course of the experiment. Correlated variables were not included in the same models. All univariate models were considered, and both backward and forward selection was used to evaluate multivariate models

Explanatory variable	Model variables	AICc	Akaike weights	*R* ^2^	*p*
Seroconversion (seronegative and infected groups)					
	Exudate in group	66.464	0.311	0.3154	.0005
	Exudate in group + ID	66.5933	0.292	0.363	.1381
	Exudate in group + change in mass	67.4307	0.192	0.3471	.229
	Exudate in pen + change in mass + ID	67.8765	0.154	0.3902	.2571
	Exudate in pen + ID	70.0439	0.052	0.295	.1171
Possible predictors	Identity (ID)^a^; treatment^b^; change in mass; number with exudate in pen (exudate in pen); number with exudate in group (exudate in group)				
Associated variables (not included in the same model)	Treatment & exudate in group; treatment & exudate in pen; exudate in group & exudate in pen				
Exudate (all groups)					
	Total seropositive in pen	110.912	0.253	0.3067	.0001
	Total seropositive in pen + change in mass	113.227	0.226	0.308	.7742
	Total seroconversions in pen + ID + treatment	116.386	0.193	0.3768	.0205
	Total seroconversions in pen + ID + treatment + change in mass	119.271	0.167	0.3776	.8233
	Total seroconversions in pen + ID	119.883	0.162	0.243	.0659
Possible predictors	Identity (ID)^a^; treatment^b^; change in mass; total seropositive in pen; total seroconversions in pen; total seroconversion per group				
Associated variables (not included in the same model)	Total seropositive per pen & total seroconversions per pen; total seropositive per pen & ID; total seropositive per pen & treatment				

a Identity of a group refers to initial status: seronegative, infected, or seropositive.

b Treatment refers to whether a group is paired with seronegative, infected, or seropositive animals in a pen.

ANOVAs were used to compare number of animals in infected groups that developed URTD, exudate, or seroconverted when paired with seronegative or seropositive groups.

## 3 RESULTS

### 3.1 Patterns of disease

Total Ig titers at the beginning of the experiment (October 2003) were significantly lower in seronegative versus seropositive groups for total antibody (*F*_1,77_ = 84.852, *p* < .001) and IgY (*F*_1,77_ = 89.656; *p* < .001), and levels of IgM did not vary among groups (*F*_1,69_ = 1.037; *p* = .312). A total of 44 animals seroconverted (16 infected and 28 seronegative animals). The Western blot technique verified seroconversions in a randomly selected group of seroconverting animals. Western blots increased to positive at least one season prior to the consistent increase in antibody titers. Signs of URTD preceded the appearance of nasal discharge (particularly purulent exudate) and seroconversion in individuals (Figure 2) and within groups (Figure 3). A contingency analysis of numbers of each treatment (seronegative and infected groups) seroconverting by year (2003, 2004, 2005) showed no significant pattern (χ^2^ = 3.538, *p* = .171). There was a difference among treatments in the number of animals that had developed URTD in the first active season of the experiment (spring 2004) (*F*_2,43_ = 6.679; *p* = .003). Seropositive groups had higher levels of URTD than from both seronegative groups (*p* = .004) and infected groups (*p* = .011). Sex did not influence the number of individuals that seroconverted (χ^2^ = 0.616, *p* = .433).

### 3.2 Antibody responses

Seroconversions occurred anywhere within 2–20 months after experimental treatments (mean = 14.1 months). Maximum increases in total antibody titers ranged from 3.35‐ to 63.35‐fold increases (mean of an 8.49‐fold increase). Of the 44 animals that seroconverted and had more than two blood samples, 37 remained high throughout the study, five had titers that dropped to seronegative and returned to seropositive, and two had titers that returned to seronegative levels. Of initially 70 seropositive animals with more than two blood samples, only one dropped to seronegative levels. In this same group, eight tortoises experienced a secondary threefold or greater increase in antibody titers (3.6‐ to 36.6‐ fold increase). There was no difference in the magnitude of increase in antibody titers among tortoises with such a secondary response to *M. agassizii*, those with a primary response to *M. agassizii*, and tortoises with primary responses to an immunization with ovalbumin quantified in Sandmeier et al. (2012) (*F*_2,46_ = 1.082; *p* = .347).

### 3.3 Disease transmission and recrudescence in experimental groups

**Figure 4:**
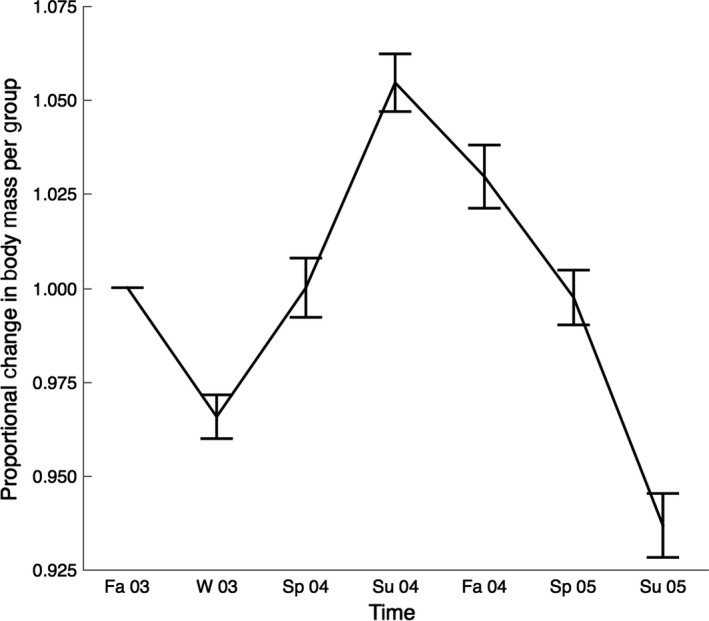
Mean proportional change in mass per group over time (mean ± standard error; *n* = 46 groups, with each group consisting of six animals). Animals were measured to an accuracy of 0.01 kg, and proportional changes in mass were calculated in relation to an animal's mass at the beginning of the experiment. Seasons are abbreviated F (fall), W (winter), Sp (spring), and Su (summer), with the corresponding last two digits of the year (2003–2005)

Mean proportional change in body mass per group did not vary among treatments (*F*_2,44_ = 1.264; *p* = .293; Figure 4). Twenty‐five animals died during the course of the study, but causes of death were unclear and not directly attributable to disease.

**Figure 5:**
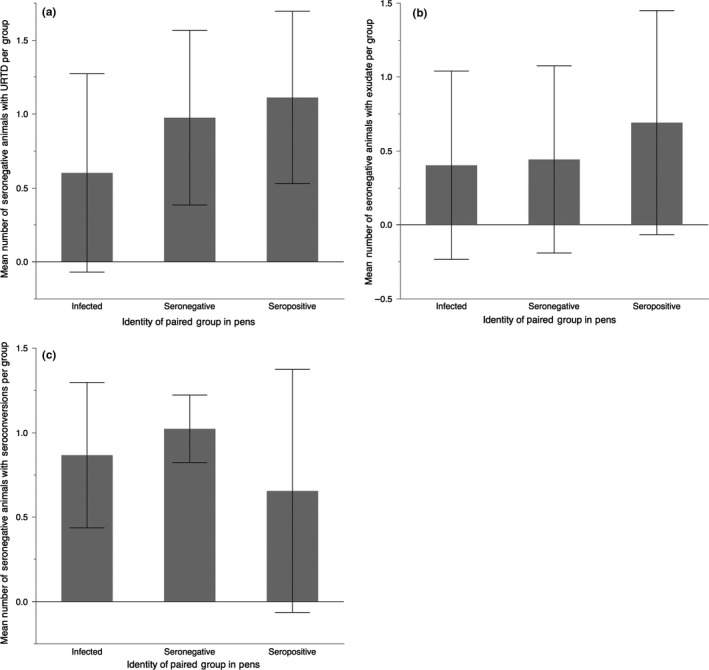
There was no statistical difference in the mean number of initially seronegative animals per group displaying disease when paired with other groups of infected, seronegative, or seropositive groups in pens (mean ± standard deviation; *n* = 17 groups, with each group consisting of six animals). Numbers of animals per group were square‐root‐transformed for normality. Disease is depicted as (a) URTD, (b) exudate, and (c) seroconversion

**Figure 6:**
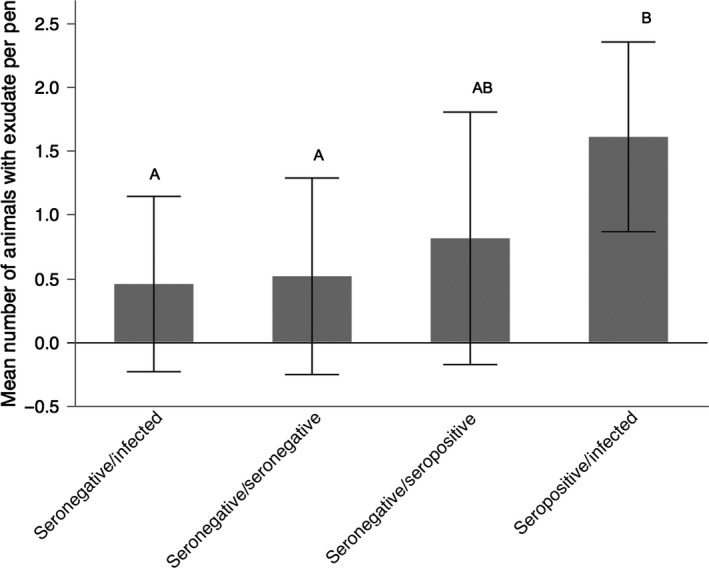
Mean number of animals with exudate per pen (mean ± standard deviation; *n* = 24 pens, with each pen consisting of 12 animals). Numbers of animals per group were square‐root‐transformed for normality. Letters indicate statistical similarity, based on Tukey's pairwise comparisons

In seronegative animals, treatment (paired with seronegative, infected, or seropositive groups) influenced neither the mean number of animals per group with URTD (*F*_2,15_ = 1.20; *p* = .33), visible exudate (*F*_2,15_ = 0.47; *p* = .64), nor the number of seroconversions (*F*_2,15_ = 0.15; *p* = .86) (Figure 5). There was a difference in the mean number of tortoises per pen that developed exudate over the course of the study in (*F*_3,42_ = 4.54; *p* = .008) (Figure 6). Only seropositive/infected pens had a greater mean number of animals that developed exudate compared to seronegative/infected pens (*p* = .010) and seronegative/seronegative pens (*p* = .020). There was no statistical difference between the other pen groupings (*p* < .05) (Figure 6).

The two best models of seroconversions per group included the number of animals with exudate in the pen and identity of the group (Table 1). By Akaike weights, these first two models are 1.62 and 1.52 times as likely as any subsequent models. The two best models of numbers of animals developing exudate per group included the total number seropositive per pen and mean change in body mass (Table 1). In both cases, the addition of the second variable to the best model did little to increase the *R* ^2^.

**Figure 7:**
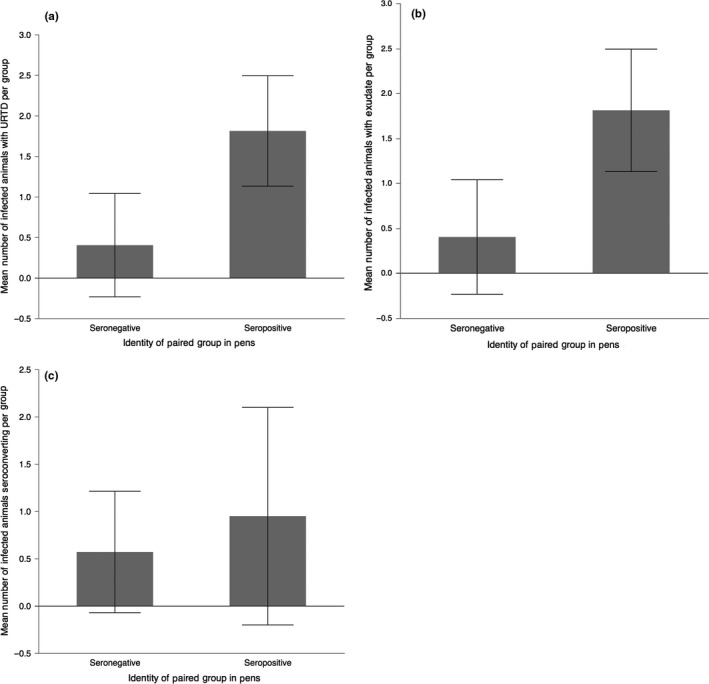
Within infected groups of tortoises, mean numbers of animals displaying disease when paired with seronegative or seropositive groups in pens (mean ± standard deviation; *n* = 12 groups of infected animals, with each group consisting of six animals). Numbers of animals per group were square‐root‐transformed for normality. More infected animals displayed signs of (a) URTD and (b) exudate when paired with seropositive animals. (c) There was no difference in the number of infected animals seroconverting, when paired with seronegative or seropositive tortoises

Infected groups of animals differed by treatment (paired with seropositive or seronegative animals) in mean levels of URTD (*F*_1,11_ = 13.70; *p* = .004) and exudate, (*F*_1,11_ = 6.47; *p* = .029), but not in mean seroconversion rates (*F*_1,11_ = 0.50; *p* = .50; Figure 7). Mean levels of exudate and URTD were lower in groups of infected animals paired with seronegative groups (Figure 7).

## 4 DISCUSSION

Recrudescence is expected to change the dynamics of host–pathogen systems in long‐lived hosts, exhibiting chronic disease and low transmission rates (e.g., Wang et al., 2013). Our study design allowed for recrudescence to be an equally important force as transmission in the prevalence of disease within groups of animals. In contrast to other transmission experiments focused on *M. agassizii* in tortoises, animals were initially free of signs of disease but allowed to interact for two full years (Aiello et al., 2016; Brown et al., 1994). While the experiment could not tease the two apart, we tested for patterns consistent with either one or both mechanisms operating on levels of disease within groups of animals. Complexities of *M. agassizii* dynamics in tortoise populations, such as apparent variation in disease progression within individual hosts, have often been ignored as background noise (Sandmeier, Tracy, DuPre, & Hunter, 2009). Here, we touch on many of these complexities and argue that they are inherently important features of this disease system. For example, mycoplasmal diseases in other organisms often cause variable severity of disease among genetically similar hosts and have load‐dependent etiologies and transmission events (Simecka et al. 1993; Stipkovits & Kempf, 1996; Waites & Talkington, 2004).

### 4.1 Patterns of disease & antibody responses

The total Ig polyclonal ELISAs (Hunter et al., 2008; Mohammadpour, 2011) used throughout this study were run with full dilution curves and gave comparable results to the light chain‐specific (total Ig) monoclonal ELISA first used to categorize animals at the beginning of the experiment (Schumacher et al., 1993; Wendland et al., 2007). Explaining some overlap among total antibody titers between groups of seropositive and seronegative animals, IgM titers were not different between the two groups of animals and likely represent natural antibody levels (Baumgarth, Tung, & Herzenberg, 2005; Hunter et al., 2008; Sandmeier et al., 2012, 2013).

The hypothesis that artificially infected tortoises will acquire disease earlier and at a greater rate than seronegative, uninfected animals, was not supported (Figure 2, Table 1). While experimentally infected tortoises appeared to have some earlier seroconversions, patterns of seroconversion were not significantly different between infected and uninfected, seronegative groups (Figure 2). For all groups of tortoises, the progression of disease was unexpectedly slow. Mild signs of URTD preceded purulent exudate and seroconversion (Figure 2). As even mild exudate can be interpreted as a sign of local inflammatory responses, it appears that innate immune responses precede adaptive immune responses by many months on average, but do not always lead to such adaptive responses or to the external discharge of exudate (Figure 3). The slow progression of disease seems at odds with an earlier study in which similar inoculations of *M. agassizii* strain PS6 led to much quicker establishment of disease in desert tortoises (Brown et al., 1994). One explanation is that the virulence of the strain has become attenuated in culture. Indeed, a similarly slow progression of disease was seen in a laboratory inoculation of six tortoises, in which flow cytometry was used to verify the viability of mycoplasmal cells at the time of infection (Mohammadpour, 2011). In that study, one tortoise clearly became infected with *M. agassizii* within months, but the other five tortoises did not seroconvert until more than a year postinoculation (Mohammadpour, 2011). While strain PS6 has been recognized as the most virulent strain of *M. agassizii* cultured from desert tortoises, attenuation of virulence may be common in this species and supports our hypothesis that *M. agassizii* may function as a recrudescent disease.

Almost all initially seropositive animals and seroconverting animals retained high positive antibody titers throughout the 2‐year duration of our experiment, similar to long‐lived antibody responses observed when captive desert tortoises were immunized with nonreplicating antigen (Sandmeier et al., 2012). Rates of URTD were not lower for these seropositive animals, suggesting that adaptive antibody responses are not protective of disease (Figure 2). Indeed, in other species, immune responses to mycoplasmas can exacerbate disease and this may be the case in desert tortoises as well (Figure 2; Simecka et al., 1993; Razin et al., 1998).

Our second hypothesis that tortoises do not exhibit clear immunological memory to *M.agassizii* was supported. Two defining features of immunological memory to extracellular pathogens include the production of long‐lived memory B cells and their rapid activation upon secondary exposure—leading to much higher antibody titers due to rapid production of high‐avidity antibodies (Murphy, 2014). Eight seropositive animals in this study experienced a marked, secondary increase in antibodies. We found a difference among antibody titers produced in primary versus secondary responses neither to *M. agassizii* nor to primary responses to immunization with ovalbumin, quantified in an earlier experiment (Sandmeier et al., 2012). The biology of reptiles, including an absence of lymph nodes and low metabolic rates, may predispose these ectothermic vertebrates to slow but long‐lived adaptive immune responses and a lack of immunological memory (Sandmeier & Tracy, 2014; Zimmerman, Vogel, & Bowden, 2010). This lack of strong immunological memory likely makes reptiles more prone to recrudescent diseases.

### 4.2 Disease transmission and recrudescence in experimental groups

Our third hypothesis that seronegative groups did not experience less disease then when paired with seronegative than with seropositive or infected groups was not supported (Figure 5). Thus, it appears that recrudescence is as important a process as transmission in introducing disease into a seemingly naïve population. One important caveat to interpretations from this experiment is that sensitive genetic tests, namely quantitative PCR, were not yet available to detect *M. agassizii* in seronegative, healthy‐appearing individuals (Braun et al., 2014; Sandmeier et al., 2017). Explicit information about the presence of the pathogen would have allowed for additional analyses on how pathogen load may be involved in recrudescence. However, recent data indicate that most healthy‐appearing animals that test positive for *M. agassizii* have very low loads of the pathogen, supporting our assumption that animals at the start of the experiment were carriers of pathogen (Sandmeier et al., 2017; Chava Weitzman unpublished data). The seemingly high rates of recrudescence may have been influenced by the conditions of the experiment (high density of tortoises without supplemental food and water), which unexpectedly led to a gradual loss in mass despite relatively favorable environmental conditions for free‐ranging tortoises in 2003–2005 (Figure 4; Sandmeier et al., 2013).

When comparing animals among pens, the infected–seropositive treatments had a significantly greater number of animals that developed exudate than the infected–negative and negative–negative treatments (Figure 6). However, experimental treatments in the absence of other factors were not significantly associated with measures of disease (Table 1), possibly due to unpredictable recrudescent rates within groups. Thus, it seems that any tortoise was susceptible to disease, although the trajectory of disease varied among pens.

To address our fourth hypothesis, the best models of both the development of exudate and seroconversion explained 20–30% of the variation in the data, suggesting that we did not measure all the variables most important to predicting disease (Table 1). Indeed, the best models suggest that a combination of recrudescence and transmission is at play—and possibly that the load of the pathogen is an important, but unquantified, variable in this disease system. Pathogen load is discussed in more detail below, but it has been implicated in other disease systems, yet remains understudied in wildlife populations (Briggs, Knapp, & Vredenburg, 2010; Tompkins, Dunn, Smith, & Telfer, 2011).

The best models for the number of animals per seronegative or infected group seroconverting included the number of animals in the group or the pen that developed exudate and the identity of the group (seronegative, infected, seropositive) (Table 1). This is not a surprising result, as visible exudate is thought to indicate local inflammation, to precede an induced antibody response, and to be responsible for increased transmission of the pathogen. Identity of the group added little explanatory power to the models (Table 1).

Similarly, the best models to explain the prevalence of exudate in each group included the number of seropositive animals per pen or the number of seroconversions per pen (less likely models) (Table 1). This result suggests either that transmission from seropositive animals was influential in within‐group disease dynamics, or that seropositive animals experienced more severe recrudescence. In reality, both of these mechanisms likely operate in conjunction with each other, and animals with the worst signs of URTD—discharging exudate—are also likely to be the most infectious, as is the case for other mycoplasmal diseases (Aiello et al., 2016; Stipkovits & Kempf, 1996). Importantly, the initial state of the tortoises—seronegative, infected, or seropositive—did not influence levels of disease, but the number of seropositive animals in the pen throughout the course of the experiment was associated with disease. This suggests that if seropositive animals are physiologically different from seronegative animals in the presence of *M. agassizii*, then those physiological differences can develop rapidly, such as antibody responses exacerbating mycoplasmal disease (Razin et al., 1998; Simecka et al., 1993). If antibody responses cause immunopathology, it is likely due to an increase in inflammatory mechanisms and a suppression of other, more efficient, mechanisms to reduce pathogen loads (Hurtado, 2012). Another possibility is that the microbial community in the nasal cavity changes with the introduction of high loads of *M. agassizii*. All these mechanisms could allow for increased loads of *M. agassizii* to develop rapidly due to immunopathology and weakening of the nasal mucosa and/or due to reduced competition from beneficial normal flora.

Our last hypothesis, predicting a potential dilution effect was largely supported. Infected groups of animals exhibited similar numbers of seroconversions, but had reduced levels of URTD and active discharge of exudate when they were paired with seronegative instead of seropositive groups (Figures 6 and 7). This suggests that in natural populations, a greater proportion of seronegative animals would limit the severity of disease within individuals. One explanation for this pattern is that initially seronegative animals were physiologically different from seropositive animals, with an increased ability to suppress loads of *M. agassizii*. The mechanism of this type of pathogen defense is unknown, but could include variation in innate immune function among individuals, which is substantial in captive desert tortoises (Sandmeier et al. 2016). Physiological variation may also include protection from different microbial communities in the nasal cavity (Tompkins et al., 2011). If this physiological difference in host susceptibility is great enough—regardless if it is genetically or developmentally determined—it could lead to a dilution effect, broadly defined as a reduction in disease due to biodiversity (Keesing, Holt, & Ostfeld, 2006; Ostfeld & Keesing, 2012). While the dilution effect has most often been applied to multihost systems (Begon, 2008; Norman, Bowers, Begon, & Hudson, 1999; Ostfeld & Keesing, 2000), the same basic mechanisms can reduce disease in single‐host systems with significant physiological differences among host individuals (e.g., Ostfeld & Keesing, 2012; Pearman & Garner, 2005).

An alternative mechanism to the dilution effect was suggested in models of chytridiomycosis in frog populations (*Rana mucosa*). In particular, the addition of pathogen load to models of disease dynamics was sufficient to produce realistic, widely varying prevalence of chytrid in host populations—without including heterogeneity in host resistance or pathogen virulence (Briggs et al., 2010). On the other hand, dilution effects within host species have also been proposed for another species of frog (*R. latestei*) and *Ranavirus*, and the possibility of an interaction between dilution effects and pathogen load remains unexplored (Pearman & Garner, 2005). While there are many differences between chytridiomycosis and mycoplasmal diseases, the physiological traits of both reptiles and amphibians—including weak or nonexistent immunological memory—may lead to an increased importance in pathogen load and/or host variation in the etiology and transmission of disease. Unlike chytridiomycosis—which can cause high mortality but persists in populations—*M. agassizii* seems to persist in individuals at low levels that are undetectable by the host immune system. A strategy of load‐dependent recrudescence and host variation in the development of disease is common for mycoplasmas (Simecka et al. 1992; Waites & Talkington, 2004), and it may also be a common strategy for a variety of pathogens of long‐lived ectothermic animals.

### 4.3 Implications & future directions

**Figure 8:**
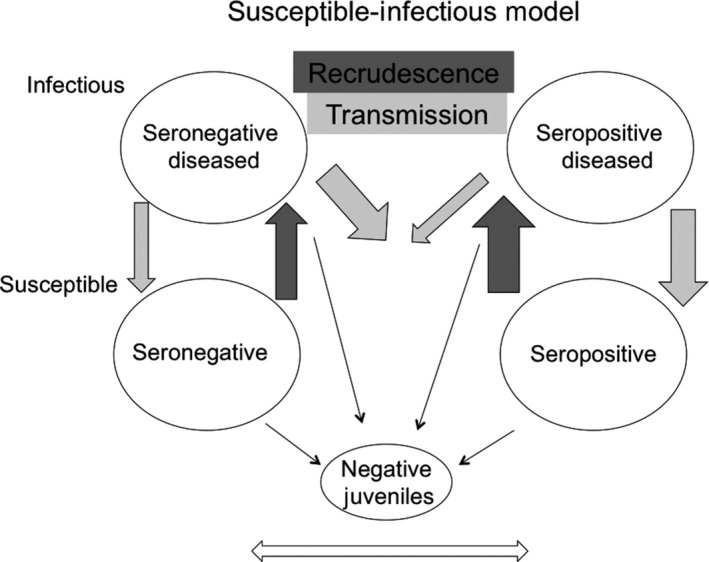
A susceptible‐infected model of mycoplasmal disease in tortoises. The ovals represent subpopulations of seronegative and seropositive infectious (“diseased”) and susceptible tortoises. Dark gray arrows represent hypothesized magnitudes of recrudescence from susceptible to infectious states. Light gray arrows represent hypothesized magnitudes of transmission of mycoplasma among groups. Black arrows represent rates of reproduction into a truly naïve, *M. agassizii*‐negative group of juveniles. The double‐sided white arrow represents the slow conversion from seronegative to seropositive and vice versa

This experiment provides support for the lack of immunological memory in desert tortoises and to the absence of a clear R state in SIR (“susceptible,” “infected,” “resistant”) models (Hudson, Rizzoli, Grenfell, Heesterbeck, & Dobson, 2003). Simple SI models will overestimate the importance of transmission in mycoplasmal URTD, and indeed, transmission has been quantified as requiring either very high loads of pathogen or long periods of contact between hosts—lasting a day or more (Aiello et al., 2016). Figure 8 depicts a conceptual SI model in which infectious animals are defined as those with the production of transferrable, external exudate and susceptible as those who are clinically healthy but likely carrying low loads of *M. agassizii*. The model explicitly depicts the heterogeneity of the adult population of tortoises, due to both chronic disease and long‐lived serological responses—allowing analyses of the effects of differing magnitudes of recrudescence and transmission rates among subgroups of tortoises. The model also depicts the low recruitment rates in populations and the importance of adults in maintaining disease prevalence. Finally, the model predicts patterns observed in this experiment, such as a large susceptible, seronegative population diluting the severity of disease—the opposite pattern that would be predicted by simple SIR models (Hudson et al., 2003).

Active management of Mojave desert tortoise populations is becoming increasingly widespread, as it is for other species of reptiles and amphibians that are facing population declines (e.g., Germano, Ewen, Mushinsky, McCoy, & Ortiz‐Catedral, 2014). As long‐lived animals, adapted to survive extreme variations in their environment, common biological markers of health—such as glucocorticoid levels and gene expression—have proven to be difficult to interpret (Bowen et al., 2015; Drake et al., 2012; O'Connor, Grumbles, George, Zimmerman, & Spotila, 1994). The prevalence of recrudescent, chronic diseases themselves may serve as an integrative biological marker of health in long‐lived species and which could prove useful in managing populations in changing environments. For example, if signs of URTD increase with stress and/or a gradual loss in mass, an increase in the prevalence of URTD in wild populations may be an indicator of more cryptic factors causing a deterioration of health.

More generally, chronic diseases in long‐lived species can have cryptic effects on population biology, patterns of which will go undetected without recognition of the importance of recrudescence in disease ecology. Indeed, a sole focus on especially virulent cases and isolated, rare associations with population declines will mischaracterize the ecological and evolutionary relationship of the host and pathogen. Recrudescence, in particular, is an unexplored mechanism that can have an equally great impact on pathogen prevalence as transmission in hosts with long life spans and weak rates of pathogen clearance and resistance.

## CONFLICT OF INTEREST

None declared.

## AUTHORS CONTRIBUTIONS

FS led the writing of the manuscript; KNM conducted all laboratory work; FS, KM, and CRT were involved in data analysis; CRT conceived and designed the project; RM and DH supported and managed all field work; HM, SD, and KH designed reagents and protocols to conduct laboratory tests and assess results. All authors gave final approval for publication, with the exception of HM, who is deceased but contributed extensively to the work prior to his death in 2016.
